# First finding of the parasitic fungus *Hesperomyces virescens* (Laboulbeniales) on native and invasive ladybirds (Coleoptera, Coccinellidae) in South Africa

**DOI:** 10.1051/parasite/2016005

**Published:** 2016-02-09

**Authors:** Danny Haelewaters, Ingrid A. Minnaar, Susana Clusella-Trullas

**Affiliations:** 1 Department of Organismic and Evolutionary Biology, Harvard University Cambridge Massachusetts USA; 2 Centre for Invasion Biology, Department of Botany and Zoology, Stellenbosch University Stellenbosch South Africa

**Keywords:** *Cheilomenes propinqua*, Coccinellidae, *Harmonia axyridis*, *Hesperomyces virescens*, Laboulbeniales

## Abstract

*Hesperomyces virescens* is a fungal ectoparasite (Laboulbeniales) that infects adult ladybirds. Research has recently focused on this parasite due to the discovery of its prevalence on the globally invasive harlequin ladybird *Harmonia axyridis* and for its potential use in studies of co-evolution and pathogen spread. We collected adults from ten species of ladybirds in the Western Cape Province, South Africa, and screened for the presence of *H. virescens*. Infections with *H. virescens* were found in the samples of two species, *H. axyridis* and the native *Cheilomenes propinqua*. This marks the first record of *H. virescens* on *H. axyridis* from the African continent and the first record on *Cheilomenes* worldwide.

## Abstract (fr)

*Hesperomyces virescens* est un ectoparasite fongique (Laboulbeniales) qui infecte les coccinelles adultes. La recherche a récemment mis l’accent sur ce parasite en raison de la découverte de sa prévalence sur la coccinelle asiatique *Harmonia axyridis*, invasive au niveau mondial, et pour son utilisation potentielle dans les études de co-évolution et de propagation des pathogènes. Nous avons recueilli les adultes de dix espèces de coccinelles dans la province du Cap occidental, Afrique du Sud et recherché la présence de *H. virescens*. Des infections à *H. virescens* ont été trouvées dans les échantillons de deux espèces, *H. axyridis* et *Cheilomenes propinqua*, une espèce indigène. Ceci est la première mention de *H. virescens* sur *H. axyridis* sur le continent africain et la première mention sur *Cheilomenes* dans le monde.

## Introduction

*Hesperomyces virescens* Thaxt. (Ascomycota: Laboulbeniomycetes: Laboulbeniales) [25] is an obligate fungal ectoparasite that infects adults of ladybirds (Coleoptera: Coccinellidae) [4]. Although most Laboulbeniales exhibit great host specificity and are restricted to a particular species or genus, *H. virescens* reportedly infects several ladybird species from around the world. It was described in 1891 by Thaxter [25] on *Chilocorus stigma* (Say, 1835) [as *C. bivulnerus*] from California, USA, and in 2002 it was found for the first time to parasitize the invasive harlequin ladybird, *Harmonia axyridis* in Ohio, USA [9]. The combination *H. virescens*-*H. axyridis* is currently widespread in the eastern United States and Western Europe, and has also been reported from the host’s native range (PR China) [11]. Only since its discovery on *H. axyridis* has *H. virescens* captured the attention of scientists. Although known for over 100 years, this fungus has been historically overlooked despite the biological and ecological importance it may have for studies of co-evolution of host and parasite.

The fungus completes its entire life cycle on the integument of a living host where individual fruiting bodies or *thalli* are formed directly from ascospores (for detailed morphology see [7]). These thalli can be formed on any part of the body of the insect [13], but germination of spores likely only starts at the moment the host cuticle has hardened [10]. The sticky spores of *H. virescens* are thought to have a short life span and are not transmitted via contact with the substrate or through air. Instead, they are spread by activities of the host [6]. Non-random distribution patterns of thalli on the body of both sexes of the host suggest that direct transmission occurs during sexual contact in the mating/feeding season [9, 14, 22, 27]. *Hesperomyces virescens* can therefore be considered a sexually transmitted disease [27].

In *H. axyridis*, which overwinters in large and dense aggregations, *H. virescens* is also socially transmitted. In the course of winter, the infection rates typically increase, suggesting that transmission through direct physical contact in overwintering aggregations is an important mechanism of spread of this fungus [17, 20, 22]. Auto-infection is caused by grooming, contributing to high thallus densities on older hosts [13, 22]. Fungus prevalence on *H. axyridis* can reach high levels, but can vary widely between locations and from season to season [13, 19].

*Harmonia axyridis* has received considerable attention because it is a striking example of a biological introduction that did not turn out as planned. Being a globally invasive alien species (IAS), its impacts are considered “immense, insidious, and usually irreversible” [15]. *Harmonia axyridis* is highly competitive with native ladybird species, and its strong dispersal capacities allow rapid range expansion into new ecosystems, hence its reputation as a devastating invader [23]. Therefore, it is important to determine which natural enemies could be used to reduce population densities of *H. axyridis*. Many regional reports have been published about parasites, parasitoids, and pathogens of *H. axyridis* both within the native and invaded range but much more could be revealed from a systematic approach [23].

In this study, we collected and screened adult individuals of ten species of ladybirds for the presence of *H. virescens* in the Western Cape Province, a region with the earliest records of established *H. axyridis* in South Africa dating from 2001 [24].

## Materials and methods

Both native and invasive ladybirds were collected from 2013 to 2015 by hand in Stellenbosch, South Africa (33°55′58.86″ S, 18°51′36.55″ E) from rose bushes planted along vineyards, oak trees, and gardens. Only adult ladybirds were collected, and specimens that had visible ectoparasitic fungal infections were not discounted during collection.

Thallus observations were made using a Leica M125 stereomicroscope, and photographs were taken using a Leica DFC320 camera and Leica Application Suite 4.0 software.

Voucher specimens of *H. virescens* were identified based on morphology [7] and are deposited at Farlow Herbarium, Harvard University, USA (FH), and Stellenbosch University Herbarium, South Africa (STEU). DNA was extracted using the Extract-N-Amp Plant PCR Kit (Sigma-Aldrich, St. Louis, MO, USA) [12] from 8 to 20 mature thalli per extraction. Sequences of SSU, ITS, and LSU ribosomal DNA (rDNA) were generated after PCR amplification using primer sets NS1/NS4, ITS1f/ITS4, and LIC24R/LR3 or LR0R/LR5, respectively [12].

## Results and discussion

Almost a third of the specimens of *H. axyridis* collected in Stellenbosch were infected by *H. virescens* while none of the other alien species showed signs of infection (Table 1). Most native species screened were not infected, except for *Cheilomenes propinqua*, which had a much lower infection rate than *H. axyridis* (Table 1).

**Table 1.** Ladybirds collected in Stellenbosch, South Africa, in the period 2013–2015, with indication of number of collected specimens (*N*) and infection prevalence (in numbers and percentages).

Lady beetle species	Status in South Africa	*N*	# infected	% infected
*Harmonia axyridis* (Pallas, 1773)	Invasive	1794	527	29.38
*Chilocorus cacti* (Linnaeus, 1767)	Introduced	263	0	0
*Hippodamia variegata* (Goeze, 1777)	Introduced	1407	0	0
*Rhyzobius lophanthae* (Blaisdell, 1892)	Introduced	36	0	0
*Cheilomenes lunata* (Fabricius, 1775)	Native	328	0	0
*Cheilomenes propinqua* (Mulsant, 1850)	Native	286	14	4.90
*Exochomus flavipes* (Thunberg, 1781)	Native	68	0	0
*Lioadalia flavomaculata* (DeGeer, 1778)	Native	36	0	0
*Oenopia cuneata* (Thunberg, 1820)	Native	344	0	0
*Psyllobora variegata* (Fabricius, 1781)	Native	30	0	0

We generated SSU, ITS, and LSU rDNA sequences of *H. virescens* taken from a South African specimen of *H. axyridis* (isolate D. Haelew. 648c, 8–10 thalli taken from the left elytron; GenBank Accession Numbers KU574863 to KU574865). We also generated SSU and LSU rDNA sequences of *H. virescens* isolated from *C. propinqua* (isolate D. Haelew. 655c, 11 thalli taken from the tip of the left elytron; GenBank Accession Numbers KU574866 and KU574867). All sequences match the already known sequences of *H. virescens* in GenBank with 96–100% similarity, which confirms our morphological identification of the fungus.

This report marks the first record of *H. virescens* on *H. axyridis* from the African continent, and the first record on *C. propinqua* worldwide. *Hesperomyces virescens* has a wide host range spanning 15 ladybird genera (following [4], given that *Hesperomyces hyperaspidis* Thaxt. was recently synonymized with *H. virescens* [1], and including the genus *Cheilomenes* Chevrolat, 1837) and is reported from all continents except Antarctica and Australia (although it is found in the Fiji Archipelago) [11, 26]. *Hesperomyces virescens* has been found on *H. axyridis* in China, the USA, Belgium, the Netherlands, Germany, United Kingdom, Czech Republic [11], Croatia [3], and most recently Hungary [18], Poland (M. Tischer, personal communication), and Slovakia (S. Viglášová, personal communication). Up to now, only two reports of *H. virescens* were known in Africa, on *Chilocorus bipustulatus* (Linnaeus, 1758) from Morocco [16] and on *Exochomus laeviusculus* Weise, 1909 from La Réunion [2].

Ceryngier and Twardowska [5] noted that the multivoltine lifestyle of *H. axyridis* likely facilitates the transmission of *H. virescens*. This is a feature shared with *Adalia bipunctata* (Linnaeus, 1758), *C. bipustulatus*, and *C. propinqua* (I.A. Minnaar, personal observation). However, *H. axyridis* combines this life-style feature with a number of other characteristics that make it the most suitable host species for *H. virescens*. *Harmonia axyridis* is highly promiscuous and overwinters in dense aggregations, supporting many more inter-generational contacts between infected and uninfected ladybirds. These characteristics provide opportunities for transmission of spores and promote the fungus to grow in large densities on individual hosts [7]. These behavioral and life history traits are likely to alter parasite-host dynamics and today *Harmonia axyridis* has become the “main host” species at least in some geographic areas, such as Belgium, the Netherlands, and parts of the eastern United States, with locally very high parasite prevalences (Table 2).

**Table 2.** Examples of studies on *Hesperomyces virescens* parasitizing *Harmonia axyridis*. The parasite prevalences (= number of infected individuals/total number of ladybirds sampled (*N*)) show that *H. axyridis* has become the “main host” species in several well-studied localities.

Country	Locality	Time	*N*	Prevalence (%)	Reference
USA	Silver Lake Farm, Kentucky	May to August 2004	147	82.3	[14]
USA	USDA-ARS, Byron, Georgia	April to October 2007	646	50.1	[21]
USA	USDA-ARS, Byron, Georgia	April 2014	306	66.4	T. Cottrell and D. Haelewaters, unpublished data
Germany	Justus-Liebig University, Giessen	January 2010	134	79.1	[5]
The Netherlands	Winterswijk, Gelderland	Winter 2010	72	55.6	[19]
Belgium	Botanic Garden Meise, Flemish Brabant	Winter 2011	86	96.5	[7]
South Africa	Stellenbosch, Western Cape	2013–2015	1794	29.4	Present paper

## Conclusion

This report supports the hypothesis that *H. virescens* is spreading around the world, possibly following *H. axyridis* as its main host. Interestingly, we also found the fungus on a native species in South Africa, *C. propinqua*, which raises the question of whether a host shift event might have taken place from the native to the invasive host following an ecological opportunity or *vice versa* [8]. These hypotheses can only be answered by undertaking global genetic analyses to assess macrogeographic population structure and reconstruct invasion pathways for both the parasite and its hosts.
